# Septic shock caused by postpartum acute pancreatitis, a case report and literature review

**DOI:** 10.1186/s12245-025-00862-y

**Published:** 2025-03-03

**Authors:** Changiz Delavari, Delaram J. Ghadimi, Maryam Taheri, Harsh Kumar, Pouya Ebrahimi, Amir Nasrollahizadeh, Sepide Javankiani

**Affiliations:** 1 Department of Plastic Surgery, Imam Khomeini Hospital of Tehran, Tehran University of Medical Sciences, Tehran, Iran; 2 School of Medicine, Shahid Beheshti University of Medical Sciences, Tehran, Iran; 3 Tehran Heart Center, Cardiovascular Diseases Research Institute, Tehran University of Medical Sciences, Tehran, Iran; 4 Liaquat National Hospital and Medical College, Karachi, Pakistan

**Keywords:** Postpartum acute pancreatitis, Biliary obstruction, Gallstones, Septic shock

## Abstract

**Introduction:**

Postpartum acute pancreatitis (PAP) is a rare but potentially life-threatening condition that can occur following childbirth. The incidence of PAP is estimated to be between 1 in 1,000 and 1 in 10,000 deliveries, with a significant proportion of cases linked to biliary causes, particularly gallstones and biliary sludge. Prompt diagnosis and comprehensive management are essential to prevent severe complications such as septic shock and peritonitis.

**Case presentation:**

We report the case of a 25-year-old white woman who presented with severe abdominal pain and septic shock 18 days after a cesarean section. Initial management included aggressive fluid resuscitation, broad-spectrum antibiotics, and pain control. Diagnostic imaging and laboratory tests confirmed the presence of biliary obstruction due to gallstones and biliary sludge, leading to acute pancreatitis. An endoscopic retrograde cholangiopancreatography (ERCP) was performed to remove the biliary obstructions, followed by a laparoscopic cholecystectomy to prevent recurrence.

**Discussion:**

PAP, while rare, poses significant risks and can lead to serious side effects such as septic shock. Early diagnosis by laboratory workup and imaging is essential. In this instance, gallstones and biliary sludge were found to be the culprit, requiring cholecystectomy and ERCP. ERCP was effective in this patient, despite its controversy in septic patients. The effective management of PAP requires a multidisciplinary approach involving obstetricians, gastroenterologists, surgeons, and critical care specialists.

**Conclusion:**

PAP must be identified and treated as soon as possible. Bile obstruction is a common problem that necessitates prompt imaging and, if necessary, endoscopic or surgical intervention. Delays can be fatal; timing is crucial. To prevent deadly consequences, doctors must be extremely suspicious of postpartum patients presenting with abdominal pain.

## Introduction

Acute pancreatitis (AP) as one the most common inflammatory pathologies of exocrine pancreas, presents rarely in the postpartum [1, 2]. The incidence of acute pancreatitis during pregnancy and the postpartum period is estimated to be between 1 in 1,000 and 1 in 10,000 births, with a significant proportion of cases occurring shortly after delivery [3, 4]. In a Sicilian cohort study, the incidence of acute pancreatitis was found to be 21.61 cases per 100,000 person-years among non-pregnant women, with a noted increase in cases during the postpartum period [5]. Pregnancy, along with four other features starting with F, fair (Caucasian), Fatty (obese), Forty (≥ 40 years), and Female, are risk factors for cholelithiasis due to estrogen level and hypoactivity of the lumen [6, 7]. Hormonal changes during pregnancy lead to increased cholesterol saturation in bile, promoting the formation of gallstones and sludge. This can obstruct the pancreatic duct and consequently cause pancreatitis [5, 8]. Hypertriglyceridemia, which can occur postpartum, is another significant risk factor for pancreatitis. Severe hypertriglyceridemia can directly induce pancreas inflammation [4, 5]. Patients usually present with abdominal pain, nausea, vomiting, and, in most cases, systemic inflammatory response, such as fever and tachycardia. If not diagnosed and managed timely, these cases can progress to septic shock, necessitating urgent medical intervention [1, 5].

Diagnosis typically involves a combination of clinical assessment and laboratory and imaging studies. Elevated serum amylase and lipase levels indicate pancreatitis. At the same time, imaging techniques such as computed tomography (CT) or magnetic resonance cholangiopancreatography (MRCP) can help visualize pancreatic inflammation and rule out other causes [1, 5, 9, 10].

The case is noteworthy because it had a rare incidence of PAP at 18 days after cesarean section, which aggravated septic shock and required immediate biliary decompression before cholecystectomy. It also draws attention to the difficulties in managing ERCP in patients who are septic and agitates the controversy around its safety in these situations. This underscores the essential role of timely diagnosis and appropriate management of PAP.

## Case presentation

A 25-year-old white woman, G1P1L1, who had undergone a cesarean section (C/S) 18 days before, was referred to the emergency room (ER) with severe abdominal pain, which had started 3 days ago. The pain was described as constant, sharp, radiating to her back, and progressive. She rated the pain intensity 8 out of 10 on the scale. The pain was aggravated by movement and deep breathing and relieved by bending forward, but it was not relieved by over-the-counter pain medications. Associated symptoms included nausea, vomiting, and significant difficulty in caring for her newborn due to the severity of the pain. Additionally, she reported not passing gas or stools for the past three days.

Her medical history was otherwise unremarkable, with no known chronic illnesses or previous abdominal surgeries other than the recent C/S. She had a healthy pregnancy with no complications until the time of delivery. There were no known allergies, and she was not taking any medications other than post-operative analgesics (Paracetamol 325 mg twice a day) prescribed after the C/S. There was no history of alcohol or drug use. Additionally, the patient was following a normal diet postpartum, with no reported high-fat intake. She was breastfeeding her newborn exclusively before her hospital admission.

Upon arrival at the ER, the patient's initial vital signs were unstable: her oxygen saturation was 93%, temperature was 39.5 °C, blood pressure was 80/undetectable pulse, heart rate was 130 beats per minute, and respiratory rate was 30 breaths per minute, indicating signs of respiratory distress. On physical examination, the patient appeared acutely ill and in distress. She had extensive abdominal distension and severe tenderness to palpation over the epigastric and lower abdomen, particularly around the C/S scar. The C/S scar appeared to be healing well, with no signs of infection, such as redness, swelling, or discharge. The remainder of the abdominal examination revealed diffuse tenderness but no rebound tenderness or guarding. Bowel sounds were absent, and there was no palpable organomegaly.

A rectal examination detected no masses, but revealed stool in the rectum, suggesting paralytic ileus rather than mechanical obstruction and the stool was heme negative. The patient's extremities were cool to the touch, with a capillary refill time of over 3 s, indicating poor peripheral perfusion. There was no peripheral edema. Neurological examination was unremarkable, with the patient being alert and oriented but in significant distress due to pain.

## Diagnostic assessment

Laboratory tests were ordered, including a complete blood count, comprehensive metabolic panel, lipase, amylase, lactate, and blood cultures. Imaging studies, including an abdominal ultrasound and a CT scan, were planned to evaluate the cause of her symptoms further and to rule out any post-operative complications such as abscess formation, bowel obstruction, or pancreatitis.

In addition to supportive care, continuous monitoring of her vital signs, electrolyte management, and serial laboratory tests, including a complete blood count, comprehensive metabolic panel, lipase, amylase, lactate, urinalysis and culture, erythrocyte sedimentation rate (ESR), C-reactive protein (CRP), and blood cultures were ordered, to track her response to the treatment and progression of her condition. The complete blood count indices revealed a high white blood cell count with a predominant percentage of neutrophils and a very low percentage of lymphocytes, indicating a significant inflammatory response. Biochemical analysis showed that total bilirubin and liver enzymes were significantly elevated. Alkaline phosphates were also elevated, which can be associated with biliary obstruction. Amylase levels were notably high, supporting the diagnosis of acute pancreatitis. Lactate dehydrogenase was significantly elevated, which can be associated with tissue damage and necrosis. Urine analysis indicated the presence of bilirubin Table 1.

**Table 1:** These laboratory findings of the patient

*Test*	*Result*	*Reference Range*
***Hematology***		
RBC (10^6^/µl)	5.58	4.2–5.5
Hemoglobin (gr/dL)	15.2	12–16
WBC (per µl)	26,300	4.000–11.000
MCV (fL)	80	80–99
Hematocrit (%)	44.6	37–47
Platelet (per µl)	159,000	150.000–400.000
Neutrophils (%)	93.6%	40–75
Lymphocytes (%)	0.9%	20–45
MCH (pg/cell)	30	27–31
MCHC (g/dL)	33	32–36
***Biochemistry and metabolic panel***		
Cholesterol	144	Up to 200
TG (mg/dl)	89	Up to 150
LDL (mg/dl)	62	Up to 130
HDL (mg/dl)	56	> 45 mg/dl
K^+^ (meq/lit)	5	3.5–5.3 meq/lit
Na(meq/lit)	142	135–148
Mg(mg/dl)	1.9	1.8–2.6
Phosphorus (mg/dl)	2.9	2.5–5
Ca (mg/dl)	8.7	8.5–10.5
Total protein (g/dl)	6	6–7.8
Albumin (g/dl)	3.6	3.5–5.2
Creatinine (mg/dl)	0.99	0.5–1.00
Urea (mg/dl)	25	13–43
Uric acid(mg/dl)	3.4	2.3–6.1
Troponin T	Negative	Negative
CK-MB(IU/L)	21	0–25
FBS (mg/dl)	82	70–115
Total Bilirubin (mg/dL)	2.9	0.1–1.2
AST (IU/L)	875	10–40
ALT (IU/L)	400	56–7
ALP (IU/L)	576	44–147
Amylase (IU/L)	4000	30–110
Lactate (mmol/L)	4.5	0.5–2.2
LDH (IU/L)	2744	140–280
ESR (mm/hr)	24	0–20
CRP (mg/L)	40	0–10
***Urine analysis***		
Urine Bilirubin	2 +	Negative
Urine Culture	Negative	Negative
***Microbiology***		
Blood Culture	Positive for E. coli, sensitive to ceftriaxone and piperacillin-tazobactam	Negative

Abbreviations: *ALP* Alkaline Phosphatase, *ALT* Alanine Aminotransferase, *AST* Aspartate Aminotransferase, *Ca* Calcium, *Cholesterol* Cholesterol, *CK-MB* Creatine Kinase-MB, *Creatinine* Creatinine, *CRP* C-Reactive Protein, *ESR* Erythrocyte Sedimentation Rate, *FBS* Fasting Blood Sugar, *HDL* High-Density Lipoprotein, *Hemoglobin* Hemoglobin, *K* + Potassium, *Lactate* Lactate, *LDH* Lactate Dehydrogenase, *LDL* Low-Density Lipoprotein, *Lymphocytes* Lymphocytes, *MCH* Mean Corpuscular Hemoglobin, *MCHC* Mean Corpuscular Hemoglobin Concentration, *MCV* Mean Corpuscular Volume, *Mg* Magnesium, *Na* Sodium, *Neutrophils* Neutrophils, *Phosphorus* Phosphorus, *Platelet* Platelet, *RBC* Red Blood Cells, *TG* Triglycerides, *Total Bilirubin* Total Bilirubin, *Urea* Urea, *Uric Acid* Uric Acid, *Urine Bilirubin* Urine Bilirubin, *WBC* White Blood Cells

**Fig. 1:**
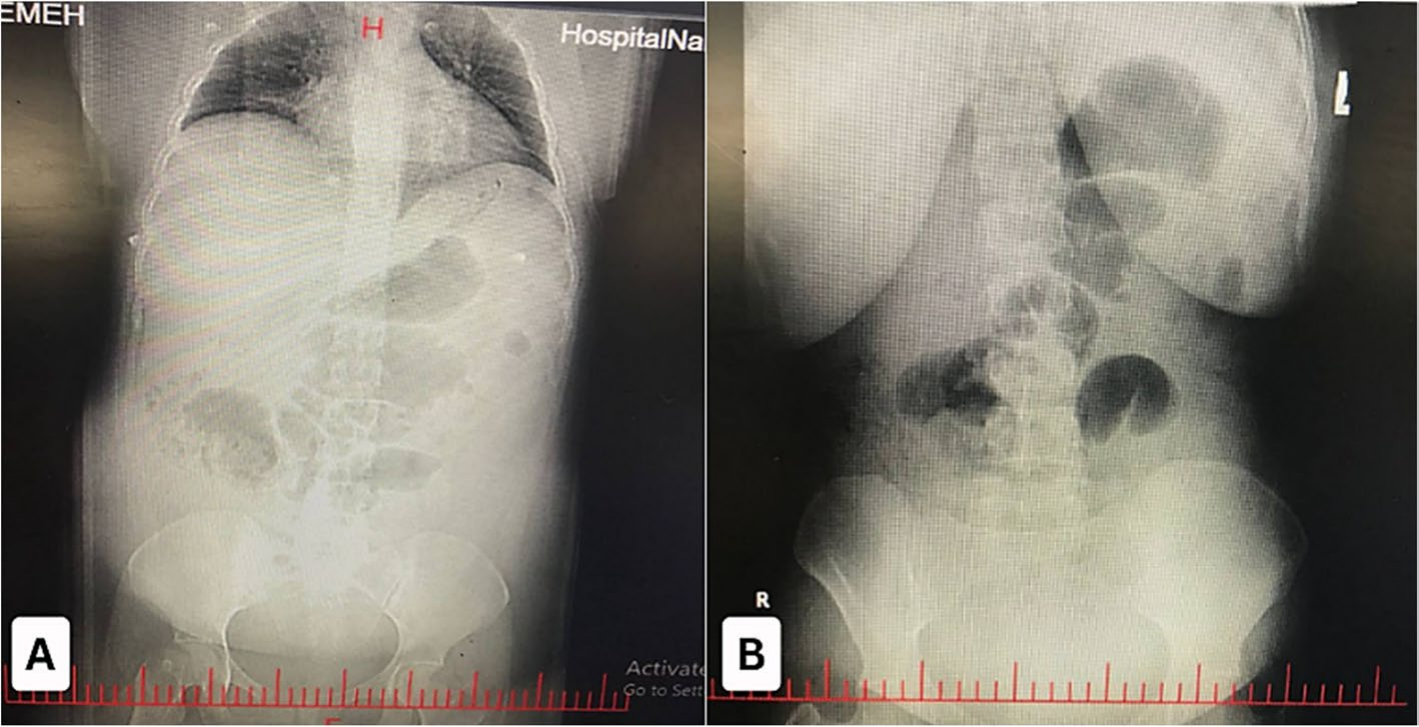
**A** Anteroposterior (AP) abdominal X-ray showing extensive dilation of the small intestine and colon loops, with no evidence of air-fluid levels or gas in the rectum, suggesting ileus. **B** Supine abdominal X-ray further illustrates the extensive dilation of the bowel loops and absence of gas in the rectum, confirming the diagnosis of ileus

A portable chest X-ray and supine and upright abdominal X-rays were ordered. Plain radiograph studies demonstrated dilation of the small intestine and colon loops, with no evidence of air-fluid levels or physical obstruction Fig. 1. Additionally, there was a lack of gas in the rectum. The abdominal X-rays also revealed multiple dilated loops of the bowel without distinct transition points, further supporting the diagnosis of ileus. There were no signs of pneumoperitoneum, ruling out gastrointestinal perforation.

Additionally, an abdominopelvic ultrasonography was performed. The bedside ultrasonographic examination revealed that the gallbladder was of normal size but exhibited increased wall thickness and layering, indicative of possible inflammation. No gallstones were detected, however, due to a brief increase in the common bile duct diameter (6.5 mm) presence of biliary sludge was suspected. A small amount of free fluid was also noted in the perihepatic region. The liver, spleen, pancreas, and kidneys appeared normal, and no other abnormalities were identified in the abdominal organs.

## Management

Two large bore intravenous (IV) access points were established. Cardiopulmonary monitoring was initiated. Oxygen was administered due to tachypnea and low oxygen saturation. Initial treatments included the administration of 2 L of normal saline over 60 min. The patient did not respond to the initial fluid therapy. Fluid resuscitation was repeated with another 2 L of normal saline over 60 min. Due to persistent hypotension, norepinephrine was initiated at a dose of 0.01 to 0.3 μg/kg/min, which improved the patient's blood pressure to 100/60 mmHg. Additionally, the patient received intravenous injections of 1 g of acetaminophen for pain management, 2 g of ceftriaxone, and 500 mg of metronidazole for broad-spectrum antibiotic coverage to address potential infectious etiologies. She was also given 40 mg of pantoprazole to reduce gastric acid secretion and prevent stress-related mucosal damage and 4 mg of ondansetron to manage nausea and vomiting.

**Fig. 2:**
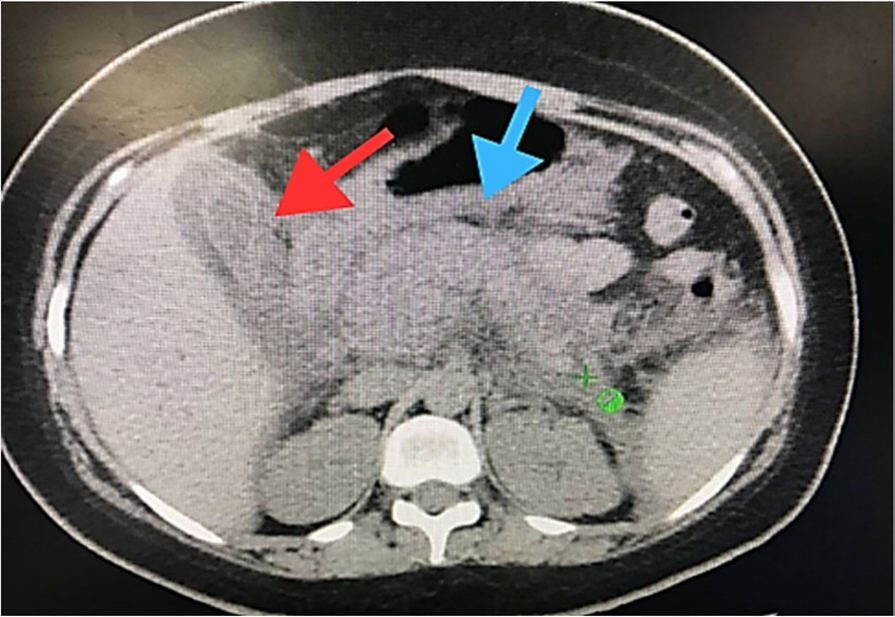
Coronal CT scan of the abdomen showing biliary sludge, gallstones (indicated by the red arrow), and evidence of pancreatitis (indicated by the blue arrow)

**Fig. 3:**
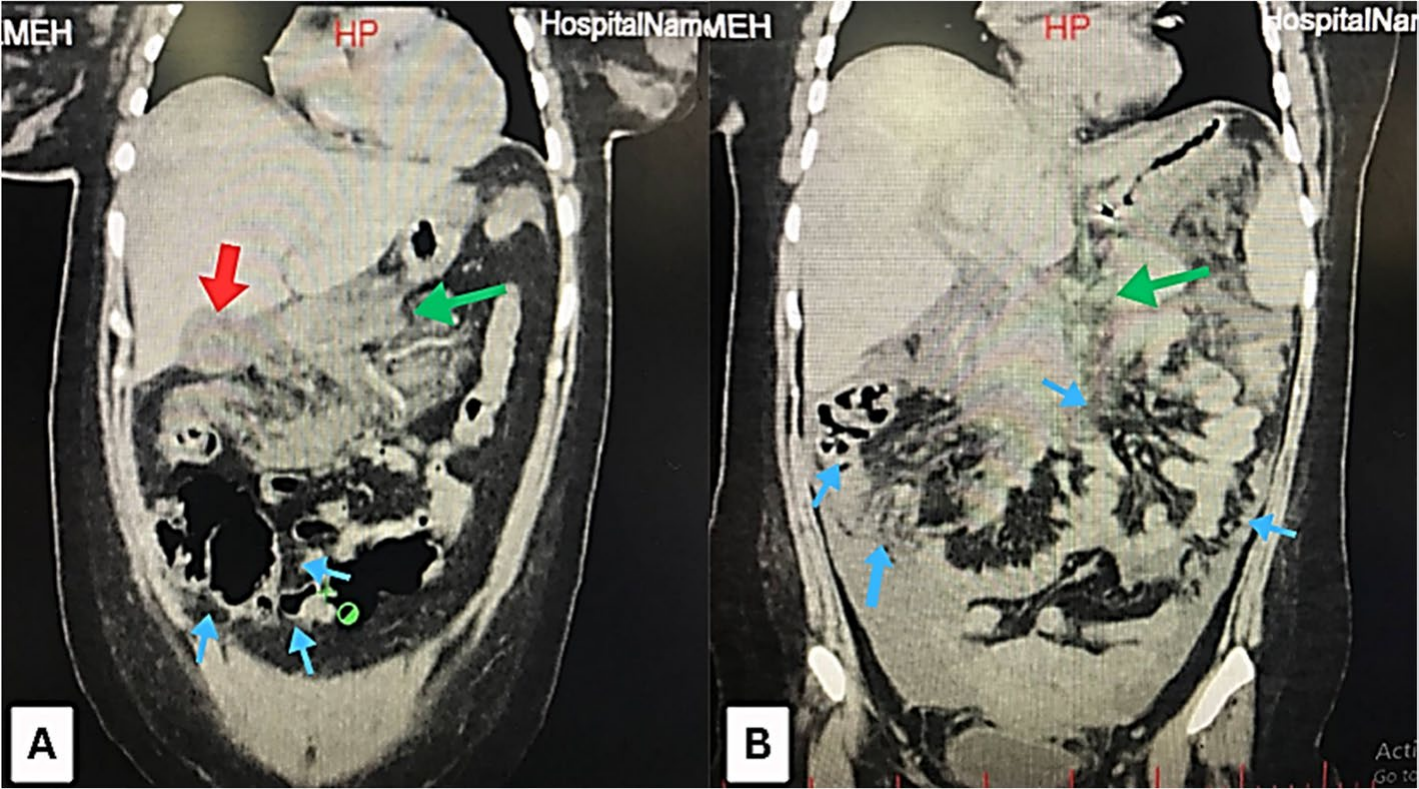
**A** An axial CT scan of the abdomen revealed gallstones (red arrow), evidence of pancreatitis (green arrow), and bowel dilation (blue arrows). **B** An axial CT scan of the abdomen showed extensive pancreatic inflammation (green arrows), additionally, (blue arrows) indicate bowel edema and peritoneal fluid accumulation, suggestive of peritonitis."

A nasogastric tube was placed to decompress the stomach and monitor gastric output, and a Foley catheter was inserted to monitor urine output and assess her fluid balance. During her hospital stay, the patient’s condition remained critical, and she was kept NPO (nil per os) to rest the pancreas and prevent further aggravation of her symptoms. A contrast-enhanced computed tomography (CECT) scan of the abdomen was performed to investigate the cause of her symptoms. The CECT scan revealed significant findings, including substantial sludge and stones in the biliary ducts, indicating obstruction. Multiple gallstones were identified, further supporting the diagnosis of biliary pancreatitis. The scan also showed evidence of acute pancreatitis, which was likely secondary to the biliary obstruction. Additionally, the presence of peritonitis, an infection of the abdominal cavity lining, was noted, further complicating her condition Figs. 2 and 3.

Considering the patient's critical condition and the diagnosis of peritonitis, the patient was transferred to the operating room under close monitoring, and anesthesia was administered. The operation commenced to resolve the biliary obstruction, alleviate the inflammation of the pancreas, and address peritonitis. The surgical procedure was crucial in preventing further deterioration of her health and ensuring her recovery. The surgical team proceeded with ERCP and laparoscopic cholecystectomy to remove the gallbladder and prevent the recurrence of biliary pancreatitis. The surgery was successful, and the patient was closely monitored postoperatively for any signs of complications.

## Follow-up

Following the operation, the patient was monitored closely. Blood culture results indicated a bacterial infection, and she was shifted to sensitive antibiotics. The combination of imaging findings and clinical presentation confirmed the diagnosis of PAP secondary to biliary obstruction complicated by peritonitis and septic shock (Septic shock was suspected based on the presence of systemic inflammatory response syndrome (SIRS), persistent hypotension despite fluid resuscitation, tachycardia, fever (39.5 °C), and leukocytosis (WBC: 26,300/μl with 93.6% neutrophils) and additionally imaging finding suggesting of peritonitis). Directed antibiotic therapy for Escherichia coli included ceftriaxone (1–2 g IV once daily) plus piperacillin-tazobactam (3.375 g IV every 6 h), adjusted based on culture sensitivity results. Additionally, due to the severity of the illness and the possibility of multidrug resistance in our patient and providing effective bacterial clearance, carbapenems (meropenem, 1-g IV q8h) were added. The presence of multiple gallstones and significant biliary sludge indicated that the biliary system was the primary source of pancreatitis. She kept NPO for over 24 h, receiving intravenous fluids for hydration. The pain was managed with Pethidine (50–100 mg IM every 4–6 h as needed) and paracetamol (1 g IV every 6 h). A gynecology and obstetrics consult revealed no abnormalities. Her condition improved steadily with this comprehensive care plan. The patient responded well to the interventions and received IV fluids and total parenteral nutrition. The patient remained NPO for 48 h and was discharged after stabilization. Her recovery was uneventful; she could drink and eat without nausea and vomiting, she had defecation, and the urinary output was proportionate with the fluid intake. During the post-operation hospital stay, no significant complications were observed. At the one-, two-, and six-week follow-ups, she reported no recurrence of abdominal pain or other symptoms. Ultrasound and laboratory tests demonstrated normal results, indicating a successful resolution of the acute episode.

## Discussion

Postpartum pancreatitis is a rare yet critical condition that necessitates timely diagnosis and management to prevent severe complications, such as septic shock [1], as observed in our patient. The septic shock caused by this condition can lead to abscess formation, necrosis, multiple organ failure, and even death if it is not promptly managed [4, 8, 9, 11]. While the incidence of AP is significantly higher in the first six months of the postpartum period compared to the pregnancy period or non-pregnant condition, cases can present earlier, as seen in this patient at 18 days postpartum. This difference is particularly associated with the higher risk of gallstone formations in these patients due to their hormonal and epidemiologic features. This suggests a notable risk for rapid deterioration in postpartum acute pancreatitis cases linked to biliary etiologies [12].

Hormonal changes, microlithiasis, which usually manifests as biliary sludge, hypertriglyceridemia, which primarily manifests as triglyceride concentrations greater than 1,000–2,000 mg/dL, and less common autoimmune pathologies can cause pancreatitis, which, if untreated, can have serious consequences like necrotizing pancreatitis [1, 6, 13, 14]. The diagnosis of PAP is confirmed by elevated liver enzymes, amylase, and lipase levels despite potential assay interference [15, 16].

The diagnosis of PAP can be challenging due to its overlap with other postpartum conditions presenting with abdominal pain, such as appendicitis, cholecystitis, and gastrointestinal perforations [17]. The absence of gallstones on ultrasonography but the presence of biliary sludge and gallbladder wall thickening were pivotal findings that directed the diagnosis toward biliary-induced pancreatitis [18, 19].

When ultrasound is inconclusive, CECT, MRCP, or endoscopic ultrasound (EUS) can provide more detailed imaging to detect microlithiasis [1, 20]. According to the World Society of Emergency Surgery (WSES) guidelines, an intraabdominal ultrasound should be performed on admission to identify the probable biliary causes. Moreover, a CECT scan can be used to confirm the diagnosis when the medical team has not reached a definite diagnosis. ERCP is considered for the removal of common bile duct stones to relieve obstruction and prevent further episodes of pancreatitis [21].

Management of PAP patients begins with stabilizing the patient's hemodynamic status through aggressive fluid resuscitation, preferably with Ringer's lactate, due to its favorable effects on systemic inflammation. Pain is managed with paracetamol, and opioids like meperidine may be used [12]. Initially, patients are kept NPO to rest the pancreas, transitioning to early enteral nutrition once stable to prevent gut barrier dysfunction [22]. A multidisciplinary approach involving obstetricians, gastroenterologists, surgeons, and critical care specialists ensures comprehensive care for both the mother and infant [4]. In addition to dietary and lipid-lowering therapies in hypertriglyceridemia-induced pancreatitis [15], further supporting evidence indicates that early conservative and advanced therapeutic intervention including transcatheter arterial embolization, can significantly improve outcomes by managing fluid collections effectively [23–26].

The role of ERCP in septic patients is still controversial [27]. Despite being the gold standard for biliary decompression, ERCP has been demonstrated to be safe and successful in the hands of skilled endoscopists, with minimal peri-procedural mortality and excellent technical success rates in critically sick patients [28]. Patients with severe sepsis or septic shock who are not stable for ERCP, however, may benefit more from other options, such as percutaneous transhepatic biliary drainage (PTBD) [29]. In severely sick patients, PTBD has also been shown to be a successful salvage technique for biliary drainage [29].

According to the WSES guidelines, empirical broad-spectrum antibiotic therapy, such as cephalosporins (ceftriaxone) and metronidazole, should be initiated as soon as possible to prevent the progression and manage infections effectively [21, 30]. Similarly, the Surviving Sepsis Campaign guidelines emphasize the critical importance of administering broad-spectrum intravenous antibiotics within the first hour of recognizing severe sepsis or septic shock. This approach aims to optimize patient outcomes by ensuring timely and adequate antimicrobial coverage, thereby reducing the risk of mortality associated with these severe infections. Both guidelines stress the need for daily reassessment of the antimicrobial regimen to optimize efficacy, prevent resistance, avoid toxicity, and minimize costs [31].

According to the European Society for Clinical Nutrition and Metabolism (ESPEN) guidelines, enteral nutrition should be initiated within 24–72 h of admission. Early enteral nutrition has been shown to significantly reduce the risk of infections, organ failure, and mortality in patients with acute pancreatitis. In severe cases where enteral nutrition is not feasible, parenteral nutrition may be required to ensure adequate nutritional support. Continuous monitoring of nutritional status and appropriate interventions are essential for managing malnutrition and improving patient outcomes in acute pancreatitis [32]. Cholecystectomy is recommended to prevent the recurrence of biliary pancreatitis and is planned once the patient's condition stabilizes [12]​. Additionally, adopting dietary restrictions, using lipid-lowering drugs, avoiding alcohol and smoking, and also maintaining regular physical activity can contribute to preventing the recurrence of pancreatitis [33, 34].

Case reports underscore the variability in PAP presentation and management. Kim et al. reported a 35-year-old woman developed severe acute pancreatitis following a cesarean section delivery, with complications such as intra-abdominal fluid collection and gastric bleeding, which was managed through percutaneous drainage, endoscopic hemostasis, and angiographic embolization [35]. Similarly, Fukami et al. reported a 17-year-old woman who developed acute pancreatitis just two hours after an uneventful delivery, highlighting the need for aggressive treatment despite an uneventful pregnancy [4]. Additionally, Sharma et al. detailed a case of severe acute pancreatitis in the early postpartum period, which was successfully managed with conservative treatment and supportive care, further emphasizing the importance of early diagnosis and tailored management to improve clinical outcomes [8].

## Conclusion

PAP is a rare but serious condition that requires prompt and accurate diagnosis to prevent severe complications. The condition is often associated with biliary obstructions, such as gallstones and biliary sludge, exacerbated by hormonal changes during and after pregnancy. A multidisciplinary approach, including aggressive fluid resuscitation, broad-spectrum antibiotics, and appropriate endoscopic or surgical interventions, is essential for effective management. Early recognition and timely, coordinated care ensure favorable outcomes and prevent recurrence in postpartum women. This case underscores the importance of vigilant monitoring and comprehensive treatment strategies for PAP.

## Data Availability

Data will be provided by the corresponding author upon reasonable request.
